# Screening of *Lactobacillus* spp. for the prevention of *Pseudomonas aeruginosa* pulmonary infections

**DOI:** 10.1186/1471-2180-14-107

**Published:** 2014-04-27

**Authors:** Youenn Alexandre, Rozenn Le Berre, Georges Barbier, Gwenaelle Le Blay

**Affiliations:** 1 Université de Brest, EA 3882-Laboratoire Universitaire de Biodiversité et d’Écologie Microbienne (LUBEM), Faculté de Médecine, 22 avenue Camille Desmoulins, 29200 Brest, France; 2 Département de Médecine Interne et Pneumologie, CHRU La Cavale-Blanche, 29200 Brest, France; 3 Université de Brest, EA 3882-Laboratoire Universitaire de Biodiversité et d’Écologie Microbienne (LUBEM), Parvis Blaise Pascal, Technopôle Brest-Iroise, 29280 Plouzané, France; 4 Université de Brest, CNRS, IFREMER, UMR 6197-Laboratoire de Microbiologie des Environnement Extrêmes (LMEE), Institut Universitaire Européen de la Mer, Place Nicolas Copernic, Technopôle Brest-Iroise, 29280 Plouzané, France

**Keywords:** *Pseudomonas aeruginosa*, *Lactobacillus*, Probiotics, Organic acids, Biofilm formation, Elastolytic activity

## Abstract

**Background:**

*Pseudomonas aeruginosa* is an opportunistic pathogen that significantly increases morbidity and mortality in nosocomial infections and cystic fibrosis patients. Its pathogenicity especially relies on the production of virulence factors or resistances to many antibiotics. Since multiplication of antibiotic resistance can lead to therapeutic impasses, it becomes necessary to develop new tools for fighting *P. aeruginosa* infections. The use of probiotics is one of the ways currently being explored. Probiotics are microorganisms that exert a positive effect on the host’s health and some of them are known to possess antibacterial activities. Since most of their effects have been shown in the digestive tract, experimental data compatible with the respiratory environment are strongly needed. The main goal of this study was then to test the capacity of lactobacilli to inhibit major virulence factors (elastolytic activity and biofilm formation) associated with *P. aeruginosa* pathogenicity.

**Results:**

Sixty-seven lactobacilli were isolated from the oral cavities of healthy volunteers. These isolates together with 20 lactobacilli isolated from raw milks, were tested for their capacity to decrease biofilm formation and activity of the elastase produced by *P. aeruginosa* PAO1. Ten isolates, particularly efficient, were accurately identified using a polyphasic approach (API 50 CHL, mass-spectrometry and *16S*/*rpoA*/*pheS* genes sequencing) and typed by pulsed-field gel electrophoresis (PFGE). The 8 remaining strains belonging to the *L. fermentum* (6), *L. zeae* (1) and *L. paracasei* (1) species were sensitive to all antibiotics tested with the exception of the intrinsic resistance to vancomycin. The strains were all able to grow in artificial saliva.

**Conclusion:**

Eight strains belonging to *L. fermentum*, *L. zeae* and *L. paracasei* species harbouring anti-elastase and anti-biofilm properties are potential probiotics for fighting *P. aeruginosa* pulmonary infections. However, further studies are needed in order to test their innocuity and their capacity to behave such as an oropharyngeal barrier against *Pseudomonas aeruginosa* colonisation *in vivo*.

## Background

*Pseudomonas aeruginosa* is one of the most common pathogens responsible for acute respiratory infections in ventilated or immunocompromised patients, and for chronic respiratory infections in cystic fibrosis (CF) patients. Between 1975 and 2003, the frequency of hospital-acquired pneumonia caused by *P. aeruginosa* increased from 9.6% to 18.1%, this pathogen thus becoming the main cause of acute respiratory infections [1]. Mortality rates in ventilator-associated pneumonia caused by *P. aeruginosa* range from 42.1 to 87% [2]. This bacteria is also associated with over 80% of the morbidity and mortality rates in CF patients [3]. These features may be explained by the wide range of both cellular associated and extracellular virulence factors involved in the pathogenesis of *P. aeruginosa* pneumonia [4,5]. Indeed, *P. aeruginosa* is endowed with remarkable virulence factors like lipopolysaccharide, type III secretion system, pyocyanin and elastase. Moreover, it is intrinsically resistant to a large number of antibiotics and can acquire resistances to many others. *P. aeruginosa* may also form biofilms that protect it from the host immune system, while decreasing antibiotics accessibility and increasing the difficulties of eradication in CF patients particularly [6]. Therapies based on the exclusive use of antibiotics may then lead to therapeutic impasses and it is necessary to find new therapeutic options to fight *P. aeruginosa* pulmonary infections.

In this context, the use of probiotic bacteria, either as prophylactic agents for preventing or delaying pulmonary colonisation with *P. aeruginosa*, or eventually as therapeutic tool to fight *P. aeruginosa* infections, seems to be particularly attractive.

The emergence of the microbiota concept, with the accumulation of evidences that human associated microbiota play a major role in health and disease [7,8], induced a profound modification in the perception of probiotics. For long confined to the gastrointestinal tract, where most of their positive effects have been described [9], they are now covering a much broader domain of applications. Evidence is emerging that probiotics may have a primordial role in health of the oral cavity [10] or in preventing mechanically ventilated patients from ventilator associated pneumonia. Several randomized controlled trials directly exploring the role of probiotics in preventing ventilator-associated pneumonia were published [11,12]. Even if the conclusions are controversial, probably because of the heterogeneity of the used probiotic strains, of the mode of administration, of the clinical situations and of the primary endpoint, this strategy seems promising. The lack of rational work for the selection of probiotic strains adapted to the respiratory ecosystem may be also one of the reasons explaining the poor results obtained in certain studies.

In this context, the main objective of this work was the screening for putative probiotic strains active against *P. aeruginosa*. Eighty-seven lactobacilli isolates, isolated from the oral cavity or raw milk, were tested for their capacity to inhibit elastolytic activity and biofilm formation, two main virulence factors of *P. aeruginosa*. In a second step, the most active isolates were characterized with molecular and phenotypic methods, and their antibiotic resistance and growth capacity in artificial saliva were checked.

## Results

### Isolation and identification of oral lactobacilli

**Figure 1:**
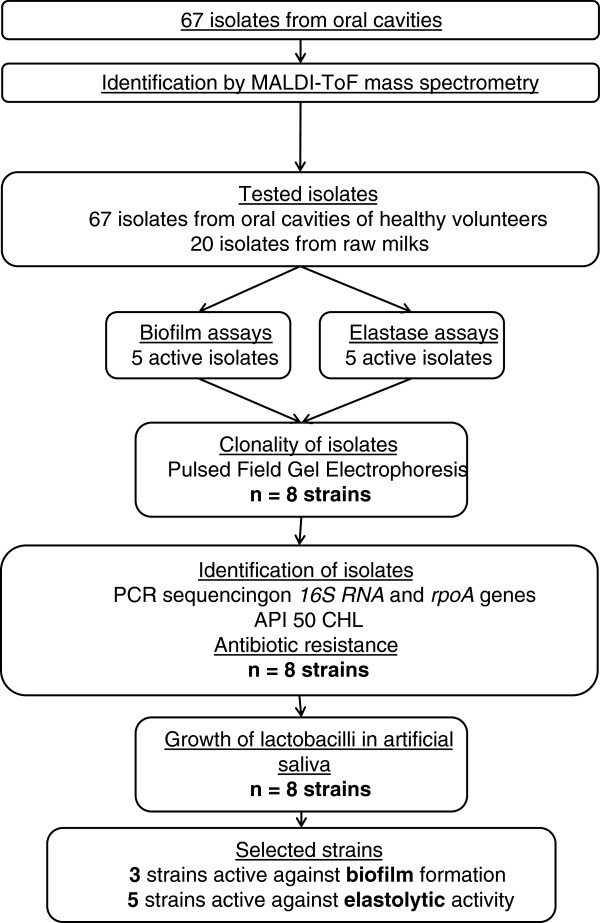
Flow-chart of the strains selection strategy.

Sixty-seven colonies were isolated from the oral cavities of 23 healthy volunteers. Among them, 7 isolates were assigned at the genus level only (*Lactobacillus*) by MALDI-TOF spectrometry analyses with scores below 1.8, whereas the majority (60 isolates) were assigned at the species level with scores above 1.8. They belonged to 9 *Lactobacillus* species (Table 1), with a strong representativeness of the *Lactobacillus reuteri* and *Lactobacillus casei* groups with respectively 40 (with a predominance of *L. fermentum*) and 21 isolates (with a predominance of *L. paracasei*). The isolation and characterization strategies of bacterial isolates are shown in Figure 1. These 67 isolates were pooled with 20 lactobacilli from a collection of bacteria isolated from raw milks [13].

**Table 1:** Preliminary identification (MALDI-TOF analyses) of newly isolated oral lactobacilli

***Lactobacillus *** groups*	Species**	**Isolates**
*L. reuteri*	*L. reuteri*	1
	*L. fermentum*	30
	*L. vaginalis*	3
*L. casei*	*L. zeae/casei*	5
	*L. rhamnosus*	6
	*L. paracasei*	10
*L. salivarius*	*L. salivarius*	4
*L. plantarum*	*L. plantarum*	1

*According to Felis and Dellaglio [14], **MALDI-ToF scores above 1.8.

### Effects of pH and acids on *P. aeruginosa* growth

*P. aeruginosa* strains are known to be sensitive to organic acids [15,16]. Since *Lactobacillus* spp. produce lactic and acetic acids that inhibit the growth of many bacteria through their undissociated forms at low pH, the sensitivity of *P. aeruginosa* PAO1 was tested toward both of them at different pH. *P. aeruginosa* PAO1 growth was monitored in LB broth by measuring OD_595nm_ values at four pH (7, 6, 5 or 4), in presence (50 or 100 mM) or absence of lactic acid. These results are presented in Table 2. *P. aeruginosa* was sensitive to the pH decrease with a pronounced growth inhibition at pH 5 and 4 (*p* < 0.0001). However, no specific effect of lactic acid was observed (*p =* 0.21*)*. The addition of acetic acid induced different effects (Table 2). As observed previously, the pH induced a significant decrease of *P. aeruginosa* (*p* < 0.0001), but in that case the addition of acetic acid induced a significant reduction of *P. aeruginosa* growth at pH 6 (from 50 mM of acetic acid and higher) and at pH 5 (from 25 mM of acetic acid and higher). No specific effect of acetic acid was detected on *P. aeruginosa* at pH 4 (no growth) nor at pH 7 (growth), whatever its concentration.

**Table 2:** ***In vitro *** **effects of pH, lactic and acetic acids on *Pseudomonas aeruginosa *** **PAO1 growth**

**Acid concentrations (mM)**	**OD**_**595nm **_**of *P. aeruginosa *** at different pH and acid concentrations*			
Lactic acid	**pH 7**	**pH 6**	**pH 5**	**pH 4**
0	0.60 ± 0.03	0.62 ± 0.01	0.43 ± 0.02	0.01 ± 0.00
50	0.74 ± 0.06	0.81 ± 0.11	0.47 ± 0.19	0.12 ± 0.01
100	0.65 ± 0.02	0.65 ± 0.02	0.23 ± 0.07	0.12 ± 0.01
Acetic acid	**pH 7**	**pH 6**	**pH 5**	**pH 4**
0	0.60 ± 0.03	0.62 ± 0.01	0.43 ± 0.02	0.01 ± 0.00
12.5	0.70 ± 0.06	0.58 ± 0.02	0.38 ± 0.27	0.12 ± 0.01
25	0.63 ± 0.06	0.65 ± 0.09	0.11 ± 0.01	0.12 ± 0.00
50	0.56 ± 0.02	0.26 ± 0.07	0.11 ± 0.01	0.12 ± 0.01
100	0.77 ± 0.14	0.10 ± 0.01	0.11 ± 0.01	0.13 ± 0.01

*Each value is the mean of triplicates ± SD (ANCOVA), *P. aeruginosa* was incubated in BHI medium for 22 h in presence of different acid concentrations (acetic, lactic acid or HCl).

### Effects of lactobacilli isolates on biofilm formation

Eighty-seven *Lactobacillus* isolates (67 from the oral cavity and 20 from raw milk) were tested for their capacity to inhibit biofilm formation by *P. aeruginosa* PAO1 (Table 3). As compared with the positive control (*P. aeruginosa* PAO1 alone), only five isolates from the oral cavity significantly (*p* < 0.05) reduced the amount of biofilm formation after 7 h of co-incubation with *P. aeruginosa* PAO1 at 37°C in BHI broth (Table 3). The four *L. fermentum* isolates ES.A.2, ES.F.115, ES.A.1a and ES.A.6a induced a biofilm reduction of 3, 7, 10 and 11% respectively, whereas *L. paracasei* ES.D.88 induced a reduction of 15%. Despite a pH decrease of the BHI medium (pH 7.4) ranging from 0.6 to 1 unit during the 7 h of co-incubation, no growth inhibition of *P. aeruginosa* was observed.

**Table 3:** ***In vitro *** **relative effects of lactobacilli isolates against biofilm formation and elastolytic activity of *Pseudomonas aeruginosa *** **PAO1**

Biofilm formation*		Elastolytic activity^*^	
*Control*	100%	*Control*	100%
*L. fermentum* ES.A.2	95%	*L. fermentum* K.C6.3.1D	63%
*L. fermentum* ES.F.115	93%	*L. zeae* Od.76	64%
*L. fermentum* ES.A.6a	88%	*L. fermentum* K.V9.3.2B	62%
*L. fermentum* ES.A.1a	88%	*L. fermentum* K.V9.3.2C	62%
*L. paracasei* ES.D.88	84%	*L. fermentum* K.C6.3.1E	53%

*Results are expressed as a percentage of biofilm formation or elastolytic activity related to control (*P. aeruginosa* PAO1 without lactobacilli). Only significantly active strains compared to control are displayed (LSD test, p < 0.05 for biofilm formation and p < 0.001 for elastolytic activity).

### Effects of lactobacilli isolates on elastolytic activity

The same eighty-seven isolates were tested for their capacity to inhibit the elastolytic activity of *P. aeruginosa* PAO1. Only five of them significantly (*p* < 0.001) reduced the activity of elastase after 22 h of co-incubation with *P. aeruginosa* PAO1 at 37°C in BHI broth, as compared with the positive control (*P. aeruginosa* PAO1 alone) (Table 3). Among them, 4 strains (K.C6.3.1D, K.V9.3.2B, K.V9.3.2C and K.C6.3.1E) were *L. fermentum* isolated from raw milk. They respectively reduced by 37%, 38%, 38% and 47% the elastolytic activity of *P. aeruginosa*. Only one strain isolated from the oral cavity (*L. zeae* Od.76), significantly reduced (minus 36%) the elastolytic activity. When grown in presence of lactobacilli during 22 h and despite a pH decrease ranging from 0.9 to 1.2 units of the BHI medium (pH 7.4), no inhibition of *P. aeruginosa* growth was observed.

### Formal identification of lactobacilli active against ***P. aeruginosa***

Once several isolates were shown to be active against *P. aeruginosa* PAO1, their identity was confirmed by *16S rRNA* and *rpoA* genes sequencing, and API 50 CHL (Table 4, Additional file 1). Two isolates (ES.D.88 and Od. 76) were identified as *L. paracasei* and *L. zeae* respectively. In the last case, it was not possible to formally distinguish between the two closely related *L. zeae* and *L. casei* species by sequencing *16S RNA* and *rpoA* genes only. The sequencing of *pheS* gene (Genbank accession number: KJ402364) was then necessary to formally assigned the Od.76 strain to the *L. zeae* species. The eight other active isolates (ES.A.1a, ES.A.2, ES.A.6a, ES.F.115, K.C6.3.1D, K.C6.3.1E, K.V9.3.2B and K.V9.3.2C) were shown to belong to *L. fermentum*. A pulsed-field gel electrophoresis (PFGE) showed that ES.A.1a, ES.A.2 and ES.A.6a were clonal strains (data not shown). ES.A.2 was then the only one kept for the last tests. The API 50 CHL gallery confirmed these results for all strains but one, *L. zeae* Od.76, since this method is not designed to identify *L. casei* nor *L. zeae*.

**Table 4:** **Species assignation of *Lactobacillus *** **isolates based on MALDI-TOF analyses, *16S RNA * and *rpoA * genes sequencing**

**Strain**	**Mass spectrometry**		***16S RNA *** **gene sequencing**		***rpoA *** **gene sequencing**		**API 50 CHL**	
	**Identification**	Score*	**Identification**	**NCBI accession number (GenBank)**	**Identification**	**NCBI accession number (GenBank)**	**Identification**	**Score**
ES.A.2	*L. fermentum*	1.916	*L. fermentum*	[KC762296]	*L. fermentum*	[KC861367]	*L. fermentum*	96.3%
ES.D.88	*L. paracasei*	2.458	*L. casei* group	[KC762297]	*L. paracasei*	[KC861369]	*L. paracasei*	98.4%
ES.F.115	*L. fermentum*	2.21	*L. fermentum*	[KC762298]	*L. fermentum*	[KC861368]	*L. fermentum*	98.5%
Od.76***	*L. zeae*	2.089	*L. casei* group	[KC762299]	*L. zeae/casei*	[KC861370]	-^**^	- ^**^
	*L. casei*	2.043						
K.C6.3.1D	*L. fermentum*	2.18	*L. fermentum*	[KC762300]	*L. fermentum*	[KC861371]	*L. fermentum*	99.7%
K.C6.3.1E	*L. fermentum*	2.109	*L. fermentum*	[KC762301]	*L. fermentum*	[KC861372]	*L. fermentum*	99.7%
K.V9.3.2B	*L. fermentum*	2.163	*L. fermentum*	[KC762302]	*L. fermentum*	[KC861373]	*L. fermentum*	99.7%
K.V9.3.2C	*L. fermentum*	2.223	*L. fermentum*	[KC762303]	*L. fermentum*	[KC861374]	*L. fermentum*	99.8%

*An isolate was considered well identified at the species level with a MALDI-TOF mass spectrometry score ≥ 1.9. **The API 50 CHL gallery is not designed to identify *L. zeae*. ***Od.76 was formally identified as a *L. zeae* by sequencing the *pheS* gene (Genbank accession number: KJ402364).

### Antibiotics sensitivity of lactobacilli

All the strains were resistant to vancomycin, as expected for lactobacilli. No other resistance was detected against the 9 other antibiotics for any of the tested strains (ampicillin, vancomycin, gentamicin, kanamycin, streptomycin, erythromycin, clindamycin, quinupristin + dalfopristin, tetracycline, chloramphenicol).

### Lactobacilli growth and acidification properties in artificial saliva

**Figure 2:**
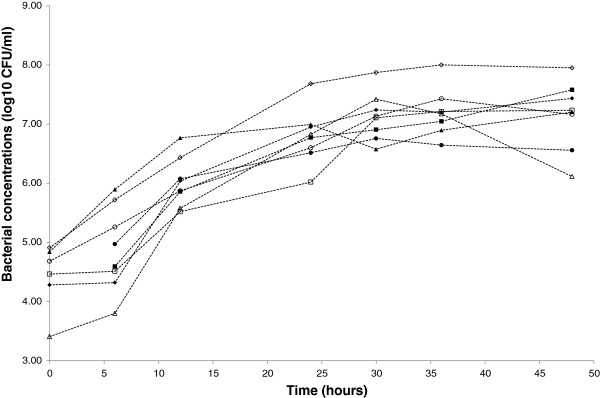
**Growth of selected strains in artificial saliva.** Bacterial concentrations (log_10_ CFU/mL) of *L. fermentum* ES.A.2 (∆), ES.F.115 (○), K.C6.3.1D (▲), K.C6.3.1E (●), K.V9.3.2B (■), K.V9.3.2C (♦), *L. zeae* Od.76 (◊) and *L. paracasei* (□). Each value is the mean of three assays.

**Figure 3:**
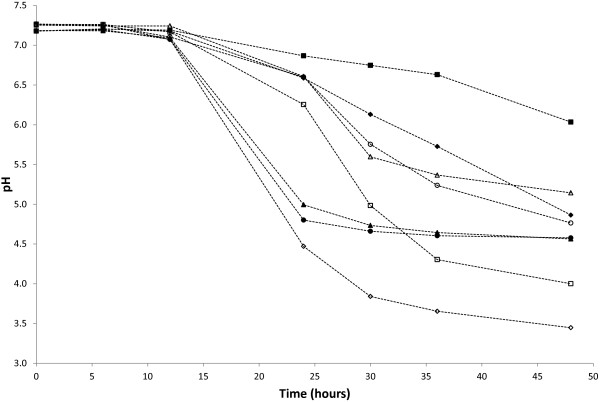
**Acidification of the artificial saliva during the growth of selected strains.** Acidifying properties of *L. fermentum* ES.A.2 (∆), ES.F.115 (○), K.C6.3.1D (▲), K.C6.3.1E (●), K.V9.3.2B (■), K.V9.3.2C (♦), *L. zeae* Od.76 (◊) and *L. paracasei* ES.D.88 (□) in artificial saliva. Each value is the mean of pH of three assays.

All the strains showed a good capacity to grow in artificial saliva with final concentrations after 48 h of incubation at 37°C comprised between 1.8 × 10^6^ and 9.3 × 10^7^ CFU/ml (Figure 2). Whatever the tested strain, no acidification occurred during the first 12 h (pH 7.14 ± 0.06) (Figure 3). The pH of artificial saliva however differed between tested strains after 24 h of incubation with a clear distinction between two groups. The first group (*L. fermentum* K.V9.3.2B, K.V9.3.2C, ES.A.2, and ES.F.115 and *L. paracasei* ES.D.88) was poorly acidifying with a decrease of 0.65 ± 0.31 pH units, whereas the second one (*L. fermentum* K.C6.3.1D, K.C6.3.1E and *L. zeae* Od.76) induced a much higher pH decrease with a diminution of 2.45 ± 0.30 units. However, after 36 h of incubation, the two facultative heterofermentative strains *L. paracasei* ES.D.88 and *L. zeae* Od.76 induced the highest pH decrease (minus 3.28 ± 0.36 pH units).

## Discussion

*P. aeruginosa* is an opportunistic pathogen in patients with significant underlying diseases. It is one of the most common causes of hospital-acquired pneumonia, especially in mechanically ventilated patients, in whom it leads to a high mortality rate [2,17]. Moreover, chronic airway inflammation with recurrent *P. aeruginosa* infections is the major cause of morbidity and mortality in patients with cystic fibrosis [18]. High incidence, infection severity and increasing resistance characterizing *P. aeruginosa* infections highlight the need for new therapeutic options. In that context, different attempts have been made to use probiotic bacteria for fighting *P. aeruginosa* pulmonary infections [19]. Lactobacilli are non-pathogenic bacteria closely associated with the human microbiota and commonly used as probiotics. Some of them are used because of their positive effects on the immune system, on the barrier effect of epithelia, whereas others are used for their capacity to fight pathogens colonisation either via competitive exclusion or antimicrobial molecules production. Probiotic effects are strain-specific, consequently they do not possess the same activity and they are not all recommended for the same health effects [20]. Specific selection criteria are then needed in order to find the right probiotic harbouring the appropriated activity (inhibition of pathogen for example) within a particular ecological niche. Some *Lactobacillus* spp. (*L. rhamnosus* GG, *L. plantarum 299, L. paracasei, L. casei, L. acidophilus*), administered by oropharyngeal application or via orogastric or nasogastric tube, have already been tested, with different levels of success, in mechanically ventilated patients to fight *P. aeruginosa* pneumonia [11,21]. To our knowledge, none of them was specifically selected according to its capacity to inhibit *P. aeruginosa*, nor to survive in the oral cavity or in the oropharynx. The main goal of this work was then to test the capacity of lactobacilli isolated from oral cavities of healthy volunteers and from raw milk to inhibit the production of virulence factors by *P. aeruginosa* PAO1 in order to look for potential probiotic bacteria capable to prevent *P. aeruginosa* pneumonia.

In this study, 67 isolates belonging to 9 *Lactobacillus* species (*L. reuteri, L. fermentum, L. vaginalis, L. rhamnosus, L. zeae, L. paracasei, L. salivarius* and *L. plantarum*), with a prevalence of *L. fermentum* and *L. paracasei*, were recovered from the oral cavities of 23 healthy volunteers. The diversity of lactobacilli isolated from the oral cavity is generally high, and these 9 species are commonly encountered in healthy persons [22-27]. Since it has been suggested that oral lactobacilli may originate from the food [28], 20 *Lactobacillus* strains (*L. fermentum, L. brevis* and *L. parabuchneri*) isolated from raw milk and whom certain species have been previously described in the oral cavity were added to increase the pool of the tested strains. Although lactobacilli do not belong to the predominant oral microbiota, in which they account for less than 1% of the cultivable fraction, they are suspected to have a considerable effect on the homeostasis of this ecosystem [29].

Among the 87 tested isolates, the 10 most active ones against *P. aeruginosa* virulence factors were identified at the species level using a polyphasic approach combining phenotypic (MALDI-TOF, API 50 CHL) and molecular (*16S rRNA* and *rpoA* genes sequencing) methods, whereas typing of *L. fermentum* strains was performed using PFGE.

Finally 8 strains (six *L. fermentum*, one *L. paracasei* and one *L. zeae*) showed a significant inhibitory effect against *P. aeruginosa* PAO1 biofilm formation or elastolytic activity. With the exception of *L. zeae* and *L. paracasei* that are facultative heterofermentative, all the active strains belonged to the *L. fermentum* species and were then obligate heterofermentative, producing both lactic and acetic acids from glucose. It has been shown that one of the major antibacterial effects of lactobacilli is mediated via lactic and acetic acids production [15]. Indeed, lactobacilli may produce high concentrations of lactic acid and acetic acid depending on their fermentative pathways and growth conditions. We have shown that *P. aeruginosa* PAO1 was sensitive to pH and acetic acid with a dose-dependent effect, growth inhibition increasing in parallel with an increase in acid concentration and pH decrease. At a pH of 4 or 5, acetic acid completely inhibited the growth of *P. aeruginosa* at a concentration of 25 mM, whereas high concentrations (≥50 mM) of acetic acid were necessary to partially inhibit *P. aeruginosa* growth at pH 6. For that reason, the inhibitory activities of lactobacilli toward *P. aeruginosa* PAO1 biofilm formation and elastolytic activity were not tested in MRS medium that contains a high glucose concentration (20 g/L), but in BHI medium. Indeed, this medium contains a low concentration of glucose (2 g/L) that limits the growth of *Lactobacillus* strains and prevents a strong acidification, allowing a better differentiation between the organic acids effects from other mechanisms of action.

Elastolytic activity and biofilm formation are two majors virulence factors observed in *P. aeruginosa*. Among the 8 strains (6 *L. fermentum*, one *L. paracasei* and one *L. zeae*) significantly inhibiting elastase activity or biofilm formation, it is interesting to note that the four *L. fermentum* strains of milk origin (*L. fermentum* K.C6.3.1D, K.C6.3.1E, K.V9.3.2B and K.V9.3.2C) inhibited elastolytic activity only, whereas the ones originating from the oral cavity (*L. fermentum* ES.A2, ES.F.115) inhibited biofilm formation only. The two other active strains from the oral cavity, *L. zeae* Od.76 and *L. paracasei* ES.D.88 significantly inhibited elastase activity and biofilm formation respectively. Elastase has been shown to destroy respiratory epithelium tight junctions, increasing permeability disorders and interleukin-8 levels while decreasing host immune response [30,31]. We previously showed in a murine model of *P. aeruginosa* pneumonia, that elastolytic activity was positively correlated to acute lung injury [5]. It has been shown by Rumbaugh *et al*. that elastolytic activity and biofilm formation are under control of the quorum sensing molecules of *P. aeruginosa*[32]. Different mechanisms of action may then be hypothesized, active *Lactobacillus* strains inhibited the quorum sensing targets*,* either they secreted antagonistic analogues of acyl-homoserine lactone or they inhibited regulating *lasR* or *lasI* genes factors [33]*.* Moreover, the use of the BHI medium that induced a limited pH decrease, together with the low number of active strains, suggested that other mechanisms of action than organic acids production were implicated. It has been shown that surface properties, such as cell charge and hydrophobicity, implicated in the non-specific adhesive capacity of bacteria differ among *Lactobacillus* strains isolated from the oral cavity of healthy volunteers, with several strains (including strains of *L. fermentum* and *L. paracasei*) showing very high adhesive properties [23]. Such a difference in surface properties between lactobacilli strains with a prevalence of high adhesive properties in lactobacilli strains isolated from the mouth might be implicated in their higher capacity to prevent biofilm formation as compared to dairy lactobacilli. Indeed surface properties are involved in adhesion properties to plastic and/or in the co-aggregation with *P. aeruginosa* that could be implicated in decreasing biofilm formation. However, further studies are needed to elucidate the antagonistic mechanism of action between described lactobacilli strains and *P. aeruginosa*.

Antagonistic activities of probiotic bacteria require a certain capacity to survive and/or to grow in the targeted ecosystem. All active strains showed a good capacity to grow in artificial saliva, suggesting that they may survive in the oral environment. However, it has been suggested that some probiotics may be implicated in the development of dental caries [34]. The use of poorly acidifying strains such as *L. fermentum* K.V9.3.2B and K.V9.3.2C inhibiting elastolytic activity and *L. fermentum* ES.F.115 and ES.A.2 inhibiting biofilm formation may then be encouraged in their use as probiotics to fight *P. aeruginosa* pulmonary infection compared to the more acidifying ones. However, knowing that acid production strongly inhibits *P. aeruginosa* growth, the use of more acidifying strains may be also investigated and subjected to an appropriate follow-up of dental health during probiotic application. Another theoretical concern regarding the safety of probiotics is the transfer of antibiotic resistance genes toward the oral and gastrointestinal microbiota. In our study, as expected for lactobacilli that are intrinsically resistant to vancomycin, all the strains were resistant to vancomycin [35]. No other resistance towards the recommended antibiotics was detected. On the other hand the toxic effect of putative probiotic on the epithelial cells from the oropharynx and respiratory tract will have to be investigated.

## Conclusions

The pathogenesis of ventilator acquired pneumonia requires micro-aspiration of oropharynx microbiota into the lower airway. The natural oropharynx microbiota of patients is modified by exogenous bacteria from the hands of the health care workers for example or by endogenous bacteria such as the intestinal microbiota by retrograde contamination. In our study, we screened 87 lactobacilli isolates from the oral cavity of healthy people and from milk with the aim to prevent *P. aeruginosa* from colonising the oropharynx environment. Eight strains, devoid of acquired antibiotic resistance were able to grow in artificial saliva and to decrease two virulence factors (elastolytic activity and biofilm formation) of *P. aeruginosa in vitro*. The next step will be to check if these strains induce a significant protective effect in an *in vivo* model of *P. aeruginosa* pneumonia.

## Methods

### Ethics

Lactobacilli from swab samples belong to the biological collection DC-2008-214 of Brest University Hospital. This biological collection was approved by Ministry of Higher Education and Research. Our study was approved by our Medical Hospital University ethics committee (« *Comité de Protection des Personnes Ouest VI »*). A written informed consent was obtained from all healthy adults volunteers participating in the study for publication of this case report. A copy of the written consent is available for review by the Editor-in-Chief of this journal. There were no under age children (<18 years-old) included in our study.

### Bacterial strains and culture conditions

*P. aeruginosa* PAO1, initially isolated from an infected wound [36], was chosen as reference strain for the activity tests. It was routinely cultivated overnight at 37°C in Luria Bertani (LB), or in Brain Heart Infusion broth (BHI) prior to the activity experiments. A total of 87 *Lactobacillus* isolates were tested for their capacity to inhibit *P. aeruginosa* PAO1 biofilm formation and elastolytic activity. Twenty of them belonged to a collection of *Lactobacillus* spp. (15 *L. fermentum*, 1 *L. brevis* and 4 *L. parabuchneri*) previously isolated from raw milks in our laboratory [13], and 67 were isolated from the mouth of healthy volunteers during this work (see below). *Lactobacillus fermentum* ATCC 9338 was obtained from AES Chemunex (Bruz, France), whereas *Lactobacillus casei* LMG 6904 and *Lactobacillus paracasei* LMG 13087 were obtained from the BCCM/LMG Bacteria Collection (http://bccm.belspo.be/about/lmg.php). They were used as reference strains for lactobacilli identification. Lactobacilli were cultured in de Mann, Rogosa and Sharpe (MRS) broth at 37°C. All strains were maintained as 33% glycerol stock at -80°C. All bacterial growth media were purchased from AES Chemunex, Bruz, France if not otherwise stated.

### Isolation and identification of oral lactobacilli

Twenty-three healthy adult volunteers were asked to rub the inside of their mouth with sterile swabs, which were then used to inoculate LAMVAB, a selective agar medium for the isolation of lactobacilli [37]. After 48 h of incubation at 37°C, single colonies were picked-up and transplanted at least thrice on MRS agar for isolation. Isolates were examined by phase-contrast microscopy, Gram stained and tested for the absence of catalase. They were then assigned to the *Lactobacillus* genus by MALDI-TOF mass spectrometry analyses with protein extraction [13]. Once several isolates were described as effective against *P. aeruginosa* PAO1, they were formally identified by sequencing their *16S rRNA*, *rpoA* and *pheS* genes, using respectively U1/RU2, rpoA-21-F/rpoA-23-R and pheS-21-F/pheS-22-R primers on their total DNA [38,39]. Part of the *16S rRNA* gene was amplified with an initial denaturation step performed for 15 min at 95°C, followed by 30 amplifications cycles consisting of 1 min at 95°C for denaturation, 30s at 64°C for primer annealing, 1 min at 72°C for extension, and one final extension of 5 min at 72°C. For the *rpoA* and *pheS* genes, an initial denaturation step was performed for 5 min at 95°C, followed by 3 amplification cycles of 1 min at 95°C, 2 min 15 s at 46°C, 1 min 15 s at 72°C, and 30 cycles of 35 s at 95°C, 1 min 15 s at 46°C, 1 min 15 s at 72°C and a final extension step of 7 min at 72°C. In few cases, an annealing temperature of 42°C was used for the amplification of *rpoA* or *pheS*. PCR amplicons were sequenced either at the molecular genetic department sequencing platform in the “CHRU de Brest” (INSERM-U1078) or at the Biogenouest platform (Roscoff, France). Sequences were then assembled using DNA Baser 3.5.3, and aligned using the MAFFT tool (http://mafft.cbrc.jp/alignment/server/) (December 2012) for species assignment. Phylogenetic analyses were conducted by the neighbour-joining method using MEGA 5.05/5.10 software [40]. Bacterial sequences for the sequenced strains have been deposited in GenBank and accession numbers are available in Table 4. Biochemical profiles were analysed by using API 50 CHL test kit (Biomérieux, Marcy l’Étoile, France). Isolates belonging to the *L. fermentum* species were then typed with PFGE analyses as described by Delavenne *et al.*[41].

### Effects of pH and organic acids on *P. aeruginosa* PAO1 growth

An overnight culture of *P. aeruginosa* PAO1 was harvested by centrifugation, washed, and suspended in the same volume of saline solution (NaCl 0.9%). One hundred microliters of this suspension was then used to inoculate 10 ml of LB supplemented with acetic acid (50 or 100 mM) or lactic acid (12.5, 25, 50 or 100 mM) at different pH (4, 5, 6 and 7). The growth of *P. aeruginosa* PAO1 was monitored by measuring the OD_600nm_ after 22 h of incubation at 37°C. Negative controls consisted in culturing *P. aeruginosa* PAO1 with no acetic or lactic acids but with hydrochloric acid (HCl) in order to reach the desired pH. All the tests were performed in triplicate.

### Inhibition of *P. aeruginosa* biofilm formation

The capacity of *Lactobacillus* isolates to inhibit biofilm formation by *P. aeruginosa* PAO1 was tested with a colorimetric method adapted from Merrit and Valdez [42,43]. *P. aeruginosa* PAO1 and lactobacilli were cultivated overnight at 37°C separately in BHI broths. After incubation, the two bacterial suspensions were washed with a saline solution, diluted in BHI (pH 7.4), and mixed in order to obtain a final suspension containing 5 × 10^7^ CFU/ml of *P. aeruginosa* and 5 × 10^7^ CFU/ml of the tested *Lactobacillus* isolate. One hundred millilitres of this bacterial suspension was deposited per well in 96-well flat-bottomed microplates (Corning Incorporated, Corning, USA), that were incubated at 37°C. After 7 h, the wells were washed twice with a saline solution (NaCl 0.9%), and 100 μl of crystal violet (0.25%) were added in each well for biofilm colouring. After 10 min, wells were washed twice again with the saline solution and the remaining crystal violet was released by addition of 100 μl of acetic acid (33%). Finally, the acetic acid solution containing the released crystal violet was transferred in a new microplate and the OD_595nm_ was measured using a spectrophotometer (Multiskan FC Microplate Photometer, Thermo Scientific, Waltham USA). The positive control was the amount of biofilm formed with a pure culture of *P. aeruginosa* PAO1, whereas the negative control was sterile BHI. Three series of four wells were performed. In parallel, the viability of *P. aeruginosa* on co-cultures with lactobacilli during 7 hours was evaluated by plate counts. Acidification was quantified through pH measurement.

### Inhibition of elastolytic activity of *P. aeruginosa*

The capacity of *Lactobacillus* isolates to inhibit the elastolytic activity of *P. aeruginosa* PAO1 was tested with a colorimetric method adapted from Rust *et al*. [44]. Aliquots (2 ml) of bacterial suspensions (*P. aeruginosa* PAO1 at 5 × 10^7^ CFU/ml and *Lactobacillus* isolate at 5 × 10^7^ CFU/ml) used for the biofilm experiment were also used in the elastase assay. They were incubated at 37°C for 22 h, centrifuged at 2 000 × *g* for 5 min, and one ml of a solution of elastin Congo-red (20 mg/ml; Sigma-Aldrich, St Louis, USA) in a 10 mM sodium phosphate buffer (pH 7.0) was added to the supernatant that was incubated at 37°C for 18 h. The insoluble elastin Congo-red was pelleted at 2 000 × *g* for 20 min and the absorbance of the Congo-red soluble fraction released by elastase, was measured at 450 nm with a spectrophotometer (Multiskan* FC Microplate Photometer, Thermo Scientific). The positive control was the elastolytic activity in a pure culture of *P. aeruginosa* PAO1 and the negative control was sterile BHI. Three series of experiments were performed. The viability of *P. aeruginosa* on co-cultures with lactobacilli during 22 hours was evaluated by plate counts. Acidification was quantified through pH measurement.

### Sensitivity of lactobacilli to antibiotics

*Lactobacillus* isolates were tested for their susceptibility to a panel of 10 antibiotics (ampicillin, vancomycin, gentamicin, kanamycin, streptomycin, erythromycin, clindamycin, quinupristin + dalfopristin, tetracycline, chloramphenicol). The screening was performed with Etests (Etest®, Biomérieux, Marcy l’Étoile, France) following instructions as indicated by the producer. Lactobacilli were cultivated on MRS agar during 48 h, colonies were picked-up and suspended in 5 ml of a saline solution (0.9% NaCl) to obtain a McFarland standard OD of 0.5, and five drops of this suspension were added to 10 ml of a new saline solution. The suspension was finally spread on blood agar plates (AES Chemunex, Bruz, France), the excess liquid was discarded, and the Etests stripes were applied to the dried plates. The minimal inhibition concentrations (MIC), expressed in mg/L, were read on the Etest stripes after 48 h of incubation at 37°C. Results were interpreted according to the cut-off levels proposed for *Enterococcus* spp. by the Committee of the antibiogram of the French society for microbiology [41,45].

### Lactobacilli growth and acidification properties in artificial saliva

The growth in artificial saliva of several *Lactobacillus* strains (*L. zeae* Od.76, *L. paracasei* ES.D.88 and *L. fermentum* ES.A.2, ES.F.115, K.C6.3.1D and K.V9.3.2C) was regularly monitored by determining the pH and plate counts on MRS agar during 48 h of incubation at 37°C. Artificial saliva composition was previously described by Roger *et al*. [46]. Initial concentrations of tested isolates were set around 5 log_10_ CFU/ml. All experiments were carried out in triplicate.

### Statistics

Statistical analyses were performed using the Microsoft Excel 2010 (Microsoft Corporation, Redmond, USA) and SAS 9.3 (SAS Institute Inc., Cary, USA) software products. In order to test the significance of the elastolytic activity and biofilm formation assays, the correlation between the three series of experiments was first assessed by the Student's inverse test (*p <* 0.05). Once the correlation between the assays was established, significant differences within each assay were carried out with a one-way analysis of variance (ANOVA), and the least significant difference (LSD) test was used to detect antagonistic activities of lactobacilli strains against *Pseudomonas aeruginosa*. Statistical significance were set at a *p* = 0.001 for elastolytic activity and *p* = 0.05 for biofilm formation. Organic acids and pH effects on *P. aeruginosa* PAO1 growth were studied by performing covariance analyses (ANCOVA) on optical densities.

## Competing interests

The authors declare they have no competing interests.

## Authors’ contributions

RLB and GLB lead the study and drafted the manuscript. YA performed the most part of the assays and drafted the manuscript. GB performed the statistics. All authors read and approved the final manuscript.

## Supplementary Material

Additional file 1 **Fermentation patterns of active strains.** This table presents the whole results obtained with the API 50 CHL gallery for the 8 tested strains. Click here for file
