# Supplementary material for: Screening of Lactobacillus spp. for the prevention of Pseudomonas aeruginosa pulmonary infections
Source: BMC Microbiol. 2014 Apr 27;14:107. doi: 10.1186/1471-2180-14-107 (PMC4040502; doi:10.1186/1471-2180-14-107)
Supplement: Additional file 1 — Fermentation patterns of active strains. This table presents the whole results obtained with the API 50 CHL gallery for the 8 tested strains. [file 1471-2180-14-107-S1.pdf]

Additional file 1: Fermentation patterns of strains active against biofilm formation or elastolytic activity

	L. fermentum						*L. paracasei*	*L. zeae*
	ES.A.2	ES.F.115	K.C6.3.1D	K.C6.3.1E	K.V9.3.2B	K.V9.3.2C	ES.D.88	Od.76
Glycerol	-	-	-	-	-	-	-	+
Erythritol	-	-	-	-	-	-	-	-
D-Arabinose	-	-	-	-	-	-	-	+
L-Arabinose	+	+	-	-	-	-	-	+
D-Ribose	+	+	+	+	+	+	+	+
D-Xylose	-	+	-	-	-	-	-	-
L-Xylose	-	-	-	-	-	-	-	-
D-Adonitol	-	-	-	-	-	-	+	-
Methyl-βD-Xylopyranoside	-	-	-	-	-	-	-	-
D-Galactose	+	+	+	+	+	+	+	+
D-Glucose	+	+	+	+	+	+	+	+
D-Fructose	+	+	+	+	+	+	+	+
D-Mannose	-	+	+	+	+	-	+	+
L-Sorbose	-	-	-	-	-	-	-	+
L-Rhamnose	-	-	-	-	-	-	-	+
Dulcitol	-	-	-	-	-	-	-	-
Inositol	-	-	-	-	-	-	+	+
D-Mannitol	-	-	-	-	-	-	+	+
D-Sorbitol	-	-	-	-	-	-	+	+
Methyl-αD-Mannopyranoside	-	-	-	-	-	-	-	-
Methyl-αD-Glucopyranoside	-	-	-	-	-	-	-	+
N-Acetylglucosamine	-	-	-	-	-	-	+	+
Amygdalin	-	-	-	-	-	-	+	+
Arbutin	-	-	-	-	-	-	+	?
Esculin ferric citrate	+	-	?	?	-	-	+	+

Salicin	-	-	-	-	-	-	+	+
D-Cellobiose	-	-	-	-	-	-	+	+
D-Maltose	+	+	+	+	+	+	+	+
D-Lactose (bovine origin)	-	+	+	+	+	+	+	+
D-Melibiose	+	+	+	+	+	+	-	-
D-Saccharose (sucrose)	+	+	+	+	+	+	+	+
D-Trehalose	+	-	+	+	-	+	+	+
Inulin	-	-	-	-	-	-	+	-
D-Melezitose	-	-	-	-	-	-	+	+
D-Raffinose	+	+	+	+	+	+	-	-
Amidon (starch)	-	-	-	-	-	-	-	+
Glycogen	-	-	-	-	-	-	-	-
Xylitol	-	-	-	-	-	-	-	-
Gentiobiose	-	-	-	-	-	-	+	+
D-Turanose	-	-	-	-	-	-	+	+
D-Lyxose	-	-	-	-	-	-	-	-
D-Tagatose	-	-	-	-	-	-	+	+
D-Fucose	-	-	-	-	-	-	-	-
L-Fucose	-	-	-	-	-	-	-	+
D-Arabitol	-	-	-	-	-	-	-	+
L-Arabitol	-	-	-	-	-	-	-	-
Potassium gluconate	-	-	?	?	-	-	-	-
Potassium 2-Ketogluconate	-	-	-	-	-	-	-	-
Potassium 5-Ketogluconate	-	-	-	-	-	-	-	-
